# Combining indoor residual spraying and insecticide-treated nets for malaria control in Africa: a review of possible outcomes and an outline of suggestions for the future

**DOI:** 10.1186/1475-2875-10-208

**Published:** 2011-07-28

**Authors:** Fredros O Okumu, Sarah J Moore

**Affiliations:** 1 Ifakara Health Institute, Biomedical and Environmental Sciences Thematic Group, P.O Box 53 Ifakara, Tanzania; 2 London School of Hygiene and Tropical Medicine, Department of Infectious and Tropical Diseases, Disease Control and Vector Biology Unit, Keppel Street, London, WC1E 7HT, UK

## Abstract

Insecticide-treated nets (ITNs) and indoor residual spraying (IRS) are currently the preferred methods of malaria vector control. In many cases, these methods are used together in the same households, especially to suppress transmission in holoendemic and hyperendemic scenarios. Though widespread, there has been limited evidence suggesting that such co-application confers greater protective benefits than either ITNs or IRS when used alone. Since both methods are insecticide-based and intradomicilliary, this article hypothesises that outcomes of their combination would depend on effects of the candidate active ingredients on mosquitoes that enter or those that attempt to enter houses. It is suggested here that enhanced household level protection can be achieved if the ITNs and IRS have divergent yet complementary properties, e.g. highly deterrent IRS compounds coupled with highly toxic ITNs. To ensure that the problem of insecticide resistance is avoided, the ITNs and IRS products should preferably be of different insecticide classes, e.g. pyrethroid-based nets combined with organophosphate or carbamate based IRS. The overall community benefits would however depend also on other factors such as proportion of people covered by the interventions and the behaviour of vector species. This article concludes by emphasizing the need for basic and operational research, including mathematical modelling to evaluate IRS/ITN combinations in comparison to IRS alone or ITNs alone.

## Background

Few vector control methods can be considered as effective against malaria mosquitoes as insecticide-treated nets (ITNs) and house spraying with residual insecticides (IRS). In recent years, endemic countries using the two methods singly or in combination have reported significant declines in malaria related morbidity and mortality [1-4]. A review of previous intervention trials has suggested that ITNs can reduce malaria cases by 39% to 62% and child mortality by 14% to 29% [5]. Similarly IRS has been shown to significantly disrupt malaria transmission, eliminate malaria vectors and reduce malaria incidence [1,6-8]

Today, universal coverage with long lasting insecticide-treated nets (LLINs) or IRS is actively promoted as the main prevention strategy under the WHO endorsed malaria control and elimination plan [9,10]. Where both ITNs and IRS are considered, the two methods are mostly used concurrently, within the same households, even though some national strategies do emphasize one method more than the other [3]. Indeed, previous and current WHO guidelines have recommended the combination of ITNs and IRS in various malaria transmission scenarios, more so for holoendemic and epidemic situations [9,11-13]. However, other than results from a small number of previous trials, which had varied primary objectives [14-16], there has not been any indisputable empirical evidence that ITN-IRS combinations can indeed offer any additional communal or personal protection, compared to using either method alone.

In this paper, recent trends of using ITNs and IRS are explored with special emphasis on: 1) significance of the two methods in current malaria control agenda, 2) potential benefits of combining the methods and 3) important research issues that should be considered to support decision making regarding combination of these two methods.

### Significance of IRS and ITNs in the current malaria control strategy

Other than intermittent preventive treatment (IPT), artemisinin-based combination therapy (ACT) and improved case detection by rapid malaria diagnostic tests (RDTs), recent declines of malaria are mostly attributable to expanded use of ITNs and IRS [2-4,17,18]. Today, these two methods remain the mainstay of malaria control agenda, a situation which is likely to continue given the remarkably slow development and adoption of alternative interventions. Therefore, while the need for new vector control tools is being addressed, one of the greatest challenges is to optimize the ongoing use of existing ITNs and IRS through evidence-based decision making, and to ensure that any accrued successes are sustained.

The current *Global Malaria Action Plan*, recently launched by the WHO-Roll Back Malaria Partnership [9], targets universal coverage of all at-risk-populations with both preventive and curative measures. The idea is to scale up preventive measures to full coverage then sustain them at that point for extended periods, thus shifting malaria control dynamics towards elimination and possibly thereafter, complete eradication. This initiative is motivated mainly by evidence that malaria morbidity and mortality has been gradually, but steadily, reducing in many countries that have well organized control programmes [3,11,19]. Regarding vector control, this new action plan primarily advocates the use of long-lasting insecticidal nets (LLINs) and IRS, and to a small extent encourages use of other methods, depending on local evidence of effectiveness. To match these targets, production, distribution and use of public health insecticides and LLINs are expected to grow exponentially. For example, it was originally approximated that 730 million LLINs would be distributed globally between 2008 and 2010, and that at least 350 million of these nets would go to Africa. In addition, 172 million households would be sprayed annually with insecticides [9].

On one hand, this new roadmap may be considered a realistic proposition given the proven effectiveness [1,4-6,20,21] and the cost-effectiveness [22,23] of the proposed methods, but also because of the gradually increasing government and donor funding for malaria control and research [3]. However, considering lessons learned from previous malaria campaigns, the targets may also be viewed as being overambitious and as exerting excessive pressure on poor malaria endemic countries, as well as on the donor community. So far even the WHO 2000 and 2005 malaria control targets [10,24,25] are yet to be met by many of these countries [3], and complete eradication is not deemed feasible in the short or medium term [26-28]. Moreover, the apparent over-reliance of the plan on insecticide-based methods is threatened by rise of insecticide resistance among target mosquito populations [29-32], which is known to have been one of the major reasons for the partial failure of malaria eradication programmes of the 1950s. Predictably, there is now a general consensus in the malaria control community that development of new vector control methods and new insecticides are key research priorities [33-37].

The WHO has provided guidelines for individual countries to use when prioritizing IRS, ITNs or both [38,39]. For example in high transmission areas, it is recommended that children and pregnant women, who are most at risk, are preferentially covered while at the same time the countries should work towards ensuring that everyone gets and uses an insecticide-treated net. Moreover, in low transmission areas, public health authorities should establish priorities based on geographical distribution of malaria [38,40]. One very significant shift from past practice is that long-lasting insecticide-treated nets (LLINs), which are designed to protect people for up to 3-5 years of use, are now being prioritized over ordinary ITNs, which have a far shorter duration of insecticidal activity [9,38]. Indeed it is expected that only LLINs will be produced in future [9]. On the other hand, IRS, which was previously recommended for use in epidemic situations, in isolated communities and in low to moderate transmission areas, is now recommended also for high transmission areas [13,39]. Perhaps most interesting, is the recognition that either ITNs or IRS if used alone may not be sufficient to disrupt malaria transmission, especially in holoendemic and hyperendemic areas, and that these two methods should preferably be combined in such situations [12,38,41].

### Combining ITNs and IRS for malaria control

#### How widespread is combined use of ITNs and IRS in Africa?

Combining ITNs and IRS for malaria control has increasingly become a common practice in Africa. At national level in sub-Saharan Africa, nearly all malaria endemic countries have adopted ITNs, IRS or both. Based on the latest world malaria report [3] more than twenty-five countries had policies involving both ITNs and IRS, including South Africa, which unlike most countries, preferentially promotes IRS over ITNs, the nets being saved for epidemic scenarios. About fifteen other countries were using ITNs but not IRS [3].

Typically, ITNs and IRS are not usually used in a mutually exclusive way. IRS is not always restricted to only households where ITNs are not already being used, and the application of IRS itself does not always preclude use of ITNs. Instead, the two methods are commonly used together in the same communities or households. For example, a common application of IRS is in the mitigation of malaria epidemics [12,13], where in many instances the residents already possess ITNs by the time IRS is launched.

Based on local evidence on malaria endemicity and other factors, such as financial costs and availability of storage and distribution systems, endemic countries often prioritize which regions should preferentially receive the different interventions. For example in Zambia, use of ITNs is targeted primarily in rural areas, while IRS is targeted primarily in urban and peri-urban areas [42], where spraying is likely to be more cost effective due to high densities of human populations. Zambia is also the only country that has ever expressly restricted mass distribution of ITNs to communities that are not eligible for IRS [43]. Nevertheless, even if promotion of IRS were restricted by government policy to areas where ITNs are not used, people may still obtain nets from the private sector or from non-governmental organizations.

#### What are the potential benefits of combining ITNs with IRS?

Despite the widespread implementation of ITNs and IRS and the likelihood of interactions between their properties, little is known about their impacts when they are used together. WHO has suggested that the two methods should be co-implemented to reduce transmission especially in hyperendemic and holoendemic scenarios [3,38]. However, these recommendations are not entirely evidence-based as very little data is available from programs where both methods have been applied, or where combined ITN/IRS interventions have been evaluated relative to either method alone. Instead, most of the data available today come from large malaria control operations conducted in communities where strategies included not only ITNs and IRS, but also other interventions including health education, artemisinin combination therapy, larviciding and environmental management [2,15,44]. Without direct measurements of transmission indicators (such as mosquito biting rates) and malaria burden indicators (such as incidence rates), from studies designed specifically to test the two vector control methods in combination, it is difficult to attribute observed protective benefits to any single intervention within the combined strategy as implemented in most of these previous large-scale interventions.

In Eritrea, where Nyarango *et al *evaluated the national malaria control programme between 2000 and 2004, there was no added advantage of using IRS and ITNs as opposed to using either method alone [44]. The authors argued that this might have been because the predominant vector in the region, *Anopheles arabiensis *was endophillic (indoor resting), and was, therefore, redundantly affected by ITNs and IRS since these interventions are both used indoors. Elsewhere, in a retrospective evaluation of control operations between 1993 and 1999 in the Solomon Islands [15], where primary malaria vectors included *Anopheles punctulatus *and the exophilic (outdoor resting), early evening feeding *Anopheles farauti *[45], it was shown that reductions in malaria and fever incidences were associated not only with DDT house spraying, but also with ITNs and health education [15]. Though this particular appraisal did not directly measure combined effects of IRS and ITNs, it was established that ITNs could not possibly replace DDT-house spraying, but that the amount of the insecticide required would be reduced if ITNs were also used.

There are also reports showing that even though combination of insecticidal nets with IRS lowered overall vector densities inside houses, there was no overall reduction in malaria transmission relative to situations where only one of the methods was used. Examples include reports by Protopopoff *et al *who evaluated the generally successful malaria control programme in the highlands of Burundi, where PermaNet 2.0™ nets, (deltamethrin treated LLINs), were deployed alongside very high coverage (90%) of deltamethrin and alpha-cypermethrin based IRS [46,47]. In this project, the interventions were targeted both spatially and temporally, so as to emphasize on areas and times when transmission was highest [46-48].

More recently, Kleinschmidt *et al *completed a review of studies involving both IRS and ITNs [14]. Of the eight previous studies that they considered, five reported a reduced risk of infection in people protected by both interventions, compared to people protected with either IRS or nets alone. This research group also analysed results of household surveys conducted between 2006 and 2008 in Bioko, Equatorial Guinea and in Zambezi province, Mozambique [14], and found that in both places, the odds of contacting malaria were significantly lower for children living in houses with both IRS and ITNs, than for children living in houses with only IRS [14].

Mathematical modelling is also increasingly being adopted as a way of estimating potential benefits of combined ITN-IRS interventions, thereby partly filling the evidence gap while awaiting controlled field trials, but also enabling informed decision making by policy makers in areas where such co-applications are already being implemented [16,41,49]. In one case, based on simulations of IRS/ITN combined interventions, Yakob *et al *[16] recently reported that even though there is likely to be significant reduction of transmission by using 80% coverage with pyrethroid treated ITNs and DDT together at household level, this combination still resulted in higher transmission potential (basic reproductive number, R_o _= 11.1 down from an control baseline of 39.5), than 80% coverage with just the ITNs alone without the DDT (R_o _= 0.1). Their explanations were that: 1) IRS compounds such as DDT, which have significant repellent properties reduce the likelihood that mosquitoes contact ITNs within the sprayed houses, and 2) ITNs prevent mosquitoes from blood feeding and, therefore, reduce the rate at which blood fed mosquitoes rest on the walls [16]. This theoretical analysis seems to undermine the protective potential of the deterrent nature of IRS insecticides and somewhat contradicts actual field results from large scale vector control evaluations which have historically shown that high coverage with IRS using DDT results in significant reduction in community malaria risk [1,6,7].

Chitnis *et al *[49] also used a mathematical model to assess effectiveness of nets and IRS (with the organochloride, DDT or a carbamate, bendiocarb) when used singly or in combination, in a holoendemic area dominated by *Anopheles gambiae*. It should be noted that whereas DDT is proven to have significant repellency against mosquitoes [50-52], bendiocarb has minimal such effects [53]. Chitnis *et al *found that humans using only ITNs are generally better protected than those with only IRS, and that even though the ITNs or IRS with DDT provided similarly high personal protection, neither of them alone could interrupt transmission on its own [49]. Besides, they also showed that high coverage of IRS using bendiocarb alone might interrupt transmission as much as simultaneous high coverage of ITNs and IRS with DDT. This finding indicates that the key question is not only whether people use IRS, ITNs or both, but that it is also imperative to consider the type of insecticides (i.e. active ingredients) used in these interventions. One other crucial suggestion from this research group was that IRS and net combinations would be most effective if the second intervention being introduced is initially targeted at those people who are not yet covered by the existing intervention [49].

Other than actual efficacy of individual insecticides, there are several other factors associated with the overall performance of these intradomicilliary interventions and their combinations. For example, a comprehensive model-based evaluation of interventions showed that in low endemicity areas, where people experience approximately three infectious mosquito bites per year (annual EIR~3) or less, LLINs alone can drive malaria transmission to levels below the 1% parasite prevalence threshold necessary to start pursuing elimination [41]. However, the same model also predicted that, in moderate transmission areas (annual EIR between 43 and 81), additional interventions such as IRS with DDT and mass screening and treatment of malaria cases, would be required alongside the LLINs to achieve the same target [41]. The situation gets more complicated when the malaria vector is more exophilic (outdoor resting) than endophillic (indoor resting). It has been suggested that in these areas and also in areas with transmission (EIR in the range of hundreds), existing interventions, even if combined, cannot completely disrupt malaria transmission [41]. As such additional interventions especially those that target outdoor-feeding or outdoor-resting mosquitoes will be required to achieve these targets [35,37,41].

Where ITN and IRS insecticides have overlapping modes of action, insecticide combinations may remain protective over longer times than in situations where only a single insecticide is used. Such an observation is exemplified in the work reported by Protopopoff *et al *in Burundi, where LLINs were provided to continue protecting people even after the residual activity of the IRS insecticides had ceased to be effective [46,47]. This concept of extending insecticide persistence can also be explained by results from studies where two different IRS insecticides were applied in same houses. In one study, Service *et al *reported that huts sprayed with both Malathion and DDT remained toxic to mosquitoes much longer and that these huts were less irritant against both *Anopheles funestus *and *An. gambiae *than huts sprayed with just DDT [52]. There are also reports from the IRS program in New Guinea in the 1950s, where pure DDT was replaced by a mixture of DDT and dieldrin in selected areas with persistently high transmission [54]. Though additional transmission reduction was observed, it could not be confirmed to be a direct result of the change of interventions. The original idea however was that the long residual effect of the DDT together with the high initial toxicity of dieldrin would be able to achieve better control of malaria than just pure DDT [54,55]. Even though existing IRS compounds last for only a few months, with the exception of DDT that lasts 6-12 months on sprayed walls [56], sustainable ITN/IRS strategies will require advanced technologies to develop long lasting formulations for IRS such as those recently tested in west Africa [36], which could achieve even greater benefits when combined with LLINs.

Based on reports analysed above, it seems that at least in some cases, there are advantages of combining ITNs with IRS relative to using either method alone, but that this outcome may be different in certain situations, since there are numerous confounding factors that can affect the results. It is therefore certain that evidence to support or refute this strategy of combinations remains inconclusive and any generalizations for optimal strategies cannot be made.

### A functional description of insecticides commonly used for IRS and ITNs, and its relevance in selecting candidate insecticides for use in combined ITN/IRS interventions

**Figure 1:**
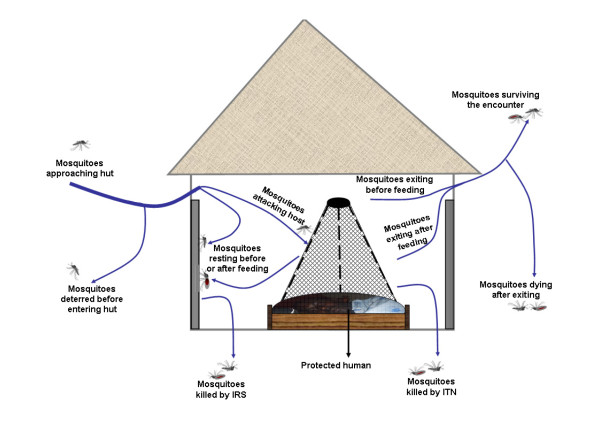
**a diagrammatic representation of various effects of ITNs and IRS on mosquitoes that enter or attempt to enter houses**. Insecticides used on nets or for IRS effect mosquitoes at different levels along the path towards the individual human inside the sprayed hut. Mosquitoes can be deterred and diverted before they enter houses, killed by the IRS or ITNs, or they can be irritated so that they exit the huts earlier than normal. Exit may occur before or after the mosquitoes have fed, but both the fed and the unfed mosquitoes may die later after they have left the huts due to sub-lethal effects of the ITN or IRS insecticides. The net and the IRS may also inhibit mosquitoes' ability to successfully take blood meals from the hut dwellers.

In practice, the decision to use IRS, ITNs or both methods should be based on existing epidemiological conditions, operational requirements and expected protective efficacy of the interventions. The protective efficacy is itself a function of several other factors including behaviour of the local mosquito populations and presence or absence of insecticide resistance among these vectors. Both IRS and ITNs are insecticide-based and they both target mosquitoes that enter or those that attempt to enter human dwellings (Figure 1). The WHO has approved 12 different insecticides for IRS and 6 for use on bed nets [56]. Two of these insecticides, deltamethrin and alpha cypermethrin can be used for both bed nets and IRS [56].

Each insecticide elicits a distinct spectrum of behavioural and physiological outcomes on mosquitoes, implying that ITNs and IRS, if based on different insecticides could differentially affect vectors even if they are simultaneously used in the same house. In this section, data from previous studies on house spraying and insecticide treated nets are considered to enable a generalised description of these interventions on the basis of how each one of them can affect mosquitoes that enter or those that attempt to enter human occupied houses (Additional files 1, 2 and 3). This functional description is then used to briefly illustrate how best one could select appropriate insecticides for a combined ITN-IRS intervention. The studies considered here were all conducted in areas with susceptible populations of anthropophilic malaria vectors *An. gambiae *and *An. funestus*, in special experimental huts designed to mimic local human houses [57].

Despite some differences in terminology [58-60], insecticides can be described generally as: 1) *deterrents or spatial repellents*, if they prevent mosquitoes from entering houses [59,61-63], 2) contact *irritants*, if they force mosquitoes that contact treated surfaces in the houses to exit, usually earlier than they normally would [59,61,64] or 3) *toxicants*, if they kill mosquitoes that contact treated surfaces or insecticide fumes [59]. In addition, insecticides may inhibit the ability of mosquitoes to take blood meals, i.e. *feeding inhibition *[65], or reduce chances of a mosquito surviving after non-lethal contacts, i.e. *sub-lethal effects *[63,66]. Computationally, deterrence or spatial repellence is calculated as the difference between number of mosquitoes entering treated huts and number entering control huts presented as a percentage of the number entering the control hut. Feeding inhibition is calculated as the percentage of all mosquitoes entering the treated huts that do not manage to feed and toxicity, as the percentage of mosquitoes entering the treated hut that die. Because in most previous studies, mosquitoes were sampled once a night as opposed to several times a night e.g. hourly, it is not possible to accurately derive values for contact irritancy based on the definition used in this article. The term excess exit is, therefore, used as a simplification for contact irritancy [59], and is calculated as the difference between percentage of mosquitoes exiting the treated huts and percentage exiting control huts.

Each of these properties is functionally applicable at different levels along the path of the mosquito, as it approaches a net-user inside an insecticide sprayed house. This process is illustrated in detail in Figure 1. Nevertheless, the properties together contribute to overall efficacy of the insecticide-based interventions. It can be argued that any interventions that reduce man vector contact and vector survival, whether by killing or by deterring host-seeking mosquitoes from potential blood sources, will subsequently also reduce the probability of mosquito-borne disease transmission [67]. Therefore even though direct toxicity has been the most desired property of public health chemicals [1], combined IRS/ITN interventions could confer superior protection against malaria at household level if the constituent applications have additional properties such as deterrence. In one example where Cullen and de Zulueta [50] were reporting on effects of DDT on malaria vectors in Uganda, they explained that the fate of mosquitoes deterred from experimental huts is intriguing in the sense that they may find food or shelter elsewhere, but also that they may die from a combination of factors such as starvation, predation and exposure to harsh environmental conditions [50]. Nevertheless, these scientists went ahead to affirm that the crucial contact between mosquitoes and humans, which is required for malaria transmission to take place between humans and mosquitoes, is reduced even without any direct toxicity [50].

Based on results outlined in Additional files 1, 2 and 3, it can be argued that while the efficacy of IRS applications is mainly due to repellency and toxicity to mosquitoes, ITNs (including LLINs) mainly inhibit feeding and kill mosquitoes. In selective cases such as when the nets are treated with permethrin, their effects can include moderate levels of repellency to the mosquitoes. It appears also that effects of insecticidal applications are augmented, moderately by their ability to inhibit blood feeding by the vectors and also the fact that they can irritate and force mosquitoes to leave houses in excess numbers. From many previous experimental hut studies, IRS with DDT or lambda cyhalothrin consistently conferred > 50% deterrence (Additional file 1, Table S1). However, bendiocarb, a carbamate commonly used for IRS, appears to be highly toxic to susceptible mosquitoes and to have significant feeding inhibition, yet it confers only limited deterrence [53,68]. This particular compound is often proposed as a potential alternative for use against insecticide resistant populations [53,68].

Insecticidal nets are effective mainly because they prevent blood feeding, even when nets become torn and also because they kill the vectors. Unlike in the case of IRS, deterrence is not a major property of LLINs (Additional file 2 Table S2). Most of the previous studies suggest that LLINs in particular elicit either very low levels of deterrence or no deterrence at all against susceptible African malaria vectors [69-74]. However, home-treated nets (also commonly referred to as conventionally treated nets) appear to consistently confer moderate levels of insecticide associated deterrence [69,72-78], even though there is one study with evidence to show that such effects may actually be due to the insecticide carrier medium and not the insecticide *per se *[77]. It is likely that IRS conveys higher deterrence than ITNs because IRS applications utilize higher quantities of insecticides, resulting in higher concentrations of the insecticide in IRS-huts than in huts containing bed nets treated with the same insecticides. This situation not withstanding, many of these previous studies also show that IRS confers only moderate feeding inhibition (Additional file 1, Table S1), and as such the intervention alone may not be adequate to prevent transmission within households. Thus, additional interventions such as nets should be incorporated to enhance personal protection at household level. Another concern regarding IRS is the rapid decay of the associated insecticidal efficacy with time. For example, while DDT-sprayed houses would not need to be re-sprayed until after 6 to 12 months, houses sprayed with pyrethroids, such as lambda cyhalothrin, must be retreated every 3-4 months to maintain acceptable efficacies [56]. Again, since this retreatment may not always be feasible, addition of LLINs is highly desirable and should be considered in such households with IRS, so that the people can continue to be protected even after the IRS insecticide has been depleted. Indeed new generation LLINs are made to last between 3-5 years and studies have now demonstrated continued efficacy of these nets after several years of use [73,74,79].

Another important element in the studies considered in Additional files 2 and 3 is the effect of wear and tear and also the effect of washing on insecticidal nets. Contrary to what may be expected, it is not clear from existing research evidence (Additional files 2, 3) that feeding inhibition is reduced when insecticidal nets are torn. It should be noted however that in most of these studies, it was not originally intended to compare torn versus intact nets, but rather the investigators used either only torn nets or only intact nets. On the other hand, while washing of nets seem to consistently reduce toxicity of conventionally treated nets, this is not the case with LLINs (Additional file 3, Table S3). Indeed there is at least one study with limited evidence to suggest that washed Olyset™ nets killed slightly more *An. gambiae *mosquitoes than unwashed nets [73] perhaps because the process of washing releases insecticide from within the net fibres to the surface where the insecticide may contact resting mosquitoes.

Lastly, variations in efficacy of IRS or nets are seemingly dependent on modes of action of actual active ingredients used. For example, considering IRS, it is clear from studies listed in Additional file 1, Table S1 that DDT has higher deterrence than both lambda cyhalothrin and bendiocarb. It can also be said that of all insecticides used in home-treated nets, permethrin appears to be the least toxic yet the most repellent and also most irritating to mosquitoes (Additional file 2, Table S2). Such differences are however not very obvious between LLINs, except that Olyset nets tend to kill fewer vectors than the other LLINs (Additional file 3, Table S3).

An important inference from this review is that toxicity to mosquitoes is not always the most significant attribute of insecticidal nets or IRS applications. There are many instances where protection is mainly due to other properties such as deterrence and feeding inhibition as opposed to simply the killing of the mosquitoes. Whereas toxic insecticidal applications arguably remain preferable in achieving mass community effects by reducing populations of biting mosquitoes [1,80-82], high coverage with repellent applications such as DDT would achieve similar community level effects by starving mosquitoes of human sources of blood, thus increasing foraging related mortality, and reducing lifetime mosquito fecundity especially in communities where there are no alternative blood hosts [6,7,83]. Thus these results also have crucial implications regarding intervention coverage and delivery systems.

This functional description can be used to improve decision-making regarding which insecticides to use when combining ITNs and IRS. Based on data from previous IRS and net applications (Additional files 1, 2 and 3), there are at least two reasons to combine the interventions. The first reason is to expand coverage and or prolong the protection even after one of the interventions is weakened, for example LLINs can be used to ensure protection long after IRS insecticides have decayed [46,47]. Similarly IRS can enhance protection in households where the nets being used are worn old, torn and have been repeatedly washed (Additional file 2, Table S2), or where some individual members of the house hold do not use the nets [84]. The second reason is to provide additional level of protection at the household level (Figure 1), for example IRS compounds with significant deterrence e.g. DDT [50,85,86] or lambda cyhalothrin [87,88] can provide an additional level of protection in households where there is a purely toxic net, or a toxic net with minimal deterrent effects e.g. PermaNet 2.0™ [69,70]. That way, effects of the combined intervention are boosted at all the stages as the mosquito approaches the net user inside sprayed house (Figure 1). Such a combination would have high deterrence (from the IRS), high mortality (from both the IRS and the ITNs) and high feeding inhibition (from the ITNs), thus significantly improving the overall effects upon vectors. If sufficiently high coverage is achieved, benefits accrued from such enhanced household level protection should lead to improved community level protection as well. Notwithstanding the argument that high deterrence could simultaneously reduce probability of mosquitoes contacting insecticides thus lowering household mortality rates and overall community benefits [16], it should be noted that in situations where mosquito vectors are highly anthropophilic e.g. *An. funestus *and *An. gambiae sensu stricto*, consistently diverting them from human dwellings, for example by spraying DDT in most dwellings in an area, has been shown to dramatically reduce vector populations and malaria transmission, as these anthropophilic vectors have few other blood sources to rely upon [1,6,7,85].

### Important research questions concerning combination of ITNs and IRS

The sections above have highlighted the fact that whereas IRS and ITNs continue to be used both singly and in combination, the current state of affairs is that it is still an open question as to whether there is any added advantage of combining the interventions. Review of previous studies has also shown that given the differences in modes of action of various IRS compounds and net types, it is likely that certain combinations may be carefully selected that result in an improved overall protection that use of either nets alone or IRS alone. But no such combinations have been experimentally compared. Conclusive evidence is therefore required to clarify the situation and allow informed decision-making. Research focusing on IRS/ITN combinations should be initiated to answer several important questions regarding the need for such combined applications. In our view, the most important of these questions are: 1) whether the two methods complement or diminish beneficial effects of each other, 2) which insecticides are the most appropriate to use in co-applications, 3) what are the epidemiological and operational determinants necessary for optimal outcomes of such co-implementation, 4) whether co-application can be used to manage challenges like insecticide resistance and finally 5) how cost-effective would the strategy be.

**Figure 2:**
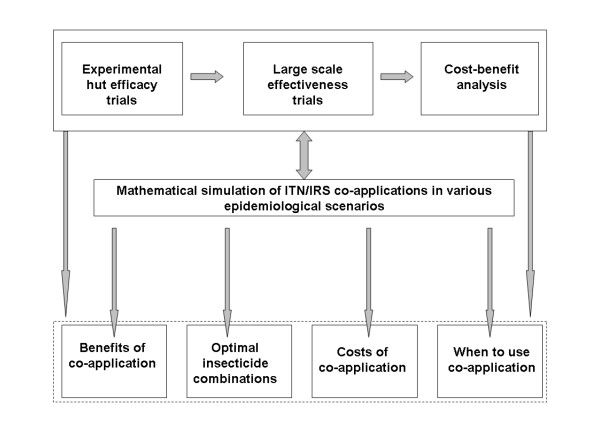
**Conceptual sequence of research necessary to generate evidence for or against combined use of ITNs and IRS**. From direct measurements in experimental hut trials, efficacious combinations of ITNs and IRS are identified and subjected to community wide effectiveness trials. Data from the effectiveness trials can then be used for cost benefit analyses. Where necessary, the mathematical models can utilize data from all the three studies (efficacy, effectiveness and cost-benefit analyses). Such simulations can: 1) help identify insecticides or combinations of insecticides for ITNs and IRS, which can then be re-evaluated in experimental huts, 2) help strengthen the design and implementation of new effectiveness trials and cost-benefit analyses and 3) enable extrapolation of information on efficacy and effectiveness of combined interventions in different epidemiological scenarios (including places with insecticide resistance). Results of these studies may then be examined to assess potential benefits of co-application, suitable insecticides for the combinations, and potential costs of the co applications as well as to determine when it is most appropriate to use the strategy.

Clearly these questions will require different kinds of studies. Therefore, research on combined ITN-IRS use should include: 1) experimental hut investigations where efficacies of the combinations are directly assessed against wild free-flying malaria vectors in malaria endemic areas, 2) mathematical simulations incorporating characteristics of candidate insecticidal applications to estimate likely benefits of the combinations in different scenarios, 3) long-term community-wide studies to determine effectiveness of the combinations and 4) cost benefit analyses of the combinations compared to individual methods on their own and also to other existing interventions. The proposed linkages between these studies are illustrated in Figure 2.

## Discussion

As malaria control enters the phase of intensive and sustained vector control, health authorities must ensure that important gains so far achieved from existing interventions are not lost. Similarly, traditional control operations must shift dynamics to reflect the current goals of malaria elimination and eradication [9], and decisions guiding these interventions should be strengthened by incorporating locally generated evidence on effectiveness. ITNs and IRS, the most widely used malaria vector control methods, are already known to confer significant benefits against malaria [1-3,5,7,8,11,17,18,44,46,47,89]. As correlations between these two methods and accrued health benefits become better understood, their acquisition and utilization also continue to expand requiring that the implementation is monitored closely to ensure proper use, optimal efficacy and maximum cost effectiveness, but also to prevent problems such as insecticide resistance and funding fatigue, as witnessed during the previous malaria eradication attempts of the 1950s and 60s [90]

The LLIN-IRS combination strategy is mostly recommended for accelerating control in high transmission areas [2,12,38,41,44], where either IRS alone or ITNs alone may not be adequate [41], but where transmission must be reduced to near-undetectable levels to achieve any significant declines in malaria prevalence [41,91-93]. However, ITNs and IRS can also be used together for different other reasons. With regards to household protection, the main reasons include ensuring protection where one of the interventions is weakened e.g. using LLINs where IRS activity decays after a short time [43,46,94] and providing additional level of protection e.g. by deterring mosquitoes from entering houses where people use toxic bed nets. However, with regards to community level protection, combinations may be used to increase overall coverage with vector control where complete coverage with only one of the interventions is unfeasible throughout all endemic communities [43]. Besides, using IRS and LLINs with differing insecticides e.g. a pyrethroid-treated LLIN and the organophosphate or carbamate IRS may slow the spread of insecticide resistance, even though there is not yet any field evidence to support this possibility. As LLINs and IRS continue to be scaled up in malaria endemic areas, the threat of insecticide resistance also increases thus management of gene mutations to the common classes of insecticides (pyrethroids, organochlorides, carbamates and organophosphates) need to be emphasised. Given that this review considers data only from sites where no insecticide resistance had been reported, it is not possible to make inferences as to how combined insecticidal applications could work in areas with high insecticide resistance. Nevertheless, it is reasonable to assume that where insecticides of different modes of action are used, mosquitoes that are resistant to one of the insecticides could still be killed by the other insecticide, thus delaying any selection for resistant mutants among the mosquito populations. The actual possibility that combinations can remain effective even where vectors are resistant to one of the active ingredients should therefore be examined urgently, preferably by way of experimental hut studies.

In the process of writing this article, it became clear that even though combining ITNs and IRS is increasingly being practiced; there is insufficient evidence as to whether it is indeed better than ITNs or IRS on their own. The article explains how different insecticides can be combined to achieve maximum benefits at household level and how this can be translated to community level protection. For example, it is argued here that IRS and ITNs can complement each other at household level, for example where the IRS power decays rapidly or where the nets are torn and repeatedly washed. It is also inferred from synthesis of several previous studies that a higher level of reduction in exposure can be achieved if highly deterrent insecticides such as DDT or lambda cyhalothrin are sprayed in houses where residents use nets treated with toxicants deltamethrin or alpha cypermethrin. The later argument is based on three principles: *a) *that any insecticide can possess an array of properties which together determine its overall protective efficacy at household level, *b) *that these properties function at different stages along the path of a mosquito approaching the human inside the house (Figure 1) and *c) *that maximizing the protective benefits at each of these stages of action is an essential process in any attempt to optimize benefits obtainable from combined ITN-IRS interventions (Figure 1). It should however be noted that this argument is particularly true in areas where the vector is still sensitive to the insecticides, but that it may not hold true in DDT/pyrethroid resistance areas. Moreover, as a cautionary measure, DDT, which is the most common organochloride, is known to be affected by the same resistance mechanism that also affects pyrethroids, both classes being amenable to target-site resistance mediated by the *kdr *gene mutation [29,36]. As such combination of DDT with pyrethroids must be very closely monitored given the likelihood of selection for more resistance without added benefit for protection. Generally, combination of pyrethroid-based IRS with any of the existing LLINs (all of which are also pyrethroid based) should be discouraged in places where there are any signs of emerging insecticide resistance, as this could lead to similar selection pressures.

Finally, to achieve community level effects, this paper recognizes the importance of coverage, i.e. proportion of all residents who consistently use these interventions, as a crucial factor. While toxic insecticidal interventions can kill large numbers of disease vectors [95-97] thus contributing to mass communal benefits, it is also noted that interventions which deter mosquitoes from potential blood-hosts and indoor resting sites also reduce the overall chances of these mosquito survival [85,98], and malaria transmission if sufficiently high coverage is achieved [1,6,7,20].

## Conclusion and recommendations

It remains largely unclear whether using both ITNs and IRS would confer significant additional benefits relative to using either method alone. Even though there have been no specific studies that expressly tested this hypothesis, previous IRS and ITN trials and a number of mathematical models have resulted in mixed results showing improved benefits in some situations and redundancy in others. Nevertheless, there are still a number of reasons that theoretically justify combination of IRS and ITNs in households. For household level protection, it is strongly recommended that where residents use pyrethroid treated LLIN, the IRS product to be sprayed in houses to supplement the nets must be of completely different mode of action. The overall epidemiological outcome of such co-applications at community level would however depend on factors such as level of intervention coverage achieved, baseline epidemiological conditions, behaviour of malaria vectors, nature of insecticides used for IRS and the type of nets being used. Therefore, to maximize any possible additional benefits from IRS/ITN co-applications, rigorous field evidence, supported by mathematical modelling where necessary, should be pursued to support the entire process of decision making, including the selection of which insecticides to be used for IRS and what type of LLINs to use.

## Conflicts of interest

The authors declare that they have no competing interests.

## Authors' contributions

FO conducted the review and drafted the manuscript. Both FO and SM wrote the final version of the manuscript. Both the authors have read and approved the final manuscript.

## Supplementary Material

Additional file 1 **Effects of insecticides commonly used for IRS in Africa**. A table showing effects of insecticides commonly used for indoor residual spraying (IRS) in Africa, on mosquitoes that enter or those that attempt to enter human occupied huts. The effects are classified as deterrence, feeding inhibition, toxicity, and excess exit. Click here for file

Additional file 2 **Properties of insecticides commonly used in ordinary home-treated ITNs**. A table showing properties of conventionally treated nets (ordinary home-treated ITNs) commonly used in Africa, on mosquitoes that enter or those that attempt to enter human huts. The effects are classified as deterrence, feeding inhibition, toxicity, and excess exit. The nets are grouped as per the active ingredients (insecticides) used to treat them. Click here for file

Additional file 3 **Properties of different long lasting insecticidal nets (LLINs) commonly used in Africa**. A table showing properties of different long lasting insecticidal nets (LLINs) commonly used in Africa, on mosquitoes that enter or those that attempt to enter human occupied huts. The effects are classified as deterrence, feeding inhibition, toxicity, and excess exit. Click here for file
