# Supplementary material for: Combining indoor residual spraying and insecticide-treated nets for malaria control in Africa: a review of possible outcomes and an outline of suggestions for the future
Source: Malar J. 2011 Jul 28;10:208. doi: 10.1186/1475-2875-10-208 (PMC3155911; doi:10.1186/1475-2875-10-208)
Supplement: Additional file 1 — Effects of insecticides commonly used for IRS in Africa. A table showing effects of insecticides commonly used for indoor residual spraying (IRS) in Africa, on mosquitoes that enter or those that attempt to enter human occupied huts. The effects are classified as deterrence, feeding inhibition, toxicity, and excess exit. [file 1475-2875-10-208-S1.DOC]

**Table S1:** Effects of insecticides commonly used for indoor residual spraying (IRS) in Africa, on mosquitoes that enter or those that attempt to enter human occupied huts. The effects are classified as deterrence, feeding inhibition, toxicity, and excess exit **^¢^**.

| **Insecticide** | **Country** | **Major vector** | **Dosage** | **Duration** | **Deterrence (%)** | **Feeding inhibition (%)** | **Toxicity (%)** | **Excess % exit** | **Reference^r^** |
| --- | --- | --- | --- | --- | --- | --- | --- | --- | --- |
| DDT | Uganda | *An. funestus* | 2g/ m^2^ WP | 7 months | 80.2 | - | 71.0 | - | [50] |
|  |  |  | 2g/ m^2^ WP | 7 months | 88.9 | - | 83.6 | - |  |
|  |  |  | 2g/ m^2^ WP | 7 months | 81.4 | - | 50.9 | - |  |
|  |  |  | 2g/ m^2^ WP | 7 months | 95.2 | - | 79.8 | - |  |
|  |  | *An. gambiae* | 2g/ m^2^ WP | 7 months | 74.4 | - | - | - | [50] |
|  |  |  | 2g/ m^2^ WP | 7 months | 89.0 | - | - | - |  |
|  |  |  | 2g/ m^2^ WP | 9 months | 69.5 | - | - | - | [85] |
|  | Nigeria | *An. gambiae* | 2g/ m^2^ WP | 6 months | 68.1 | - | 52.0 | 50.3 | [52] **^** |
|  | South Africa | *An. arabiensis* | 2g/ m^2^ WP | 3 months | 52.4 | 67.1 | 55.9 | 26.3 | [51] **^** |
|  |  |  | 2g/ m^2^ WP | 3 months | 96.6 | 0.0 | - | - | [51] **^*** |
|  |  |  | 2g/ m^2^ WP | 5 months | - | 36.0 | 32.0 | - | [88] **^** |
|  | Tanzania | *An. gambiae* | 2g/ m^2^ WP | 5 months | 56.4 | 35.1 | 17.0 | 46.2 | [86] ^+^ |
|  | Kenya | *An. gambiae* | 2g/ m^2^ WP | 6 months | 50.3 | 13.9 | 15.7 | 32.3 |  |
|  |  | *An. funestus* | 2g/ m^2^ WP | 6 months | 36.3 | 22.1 | 41.2 | 8.75 |  |
|  |  |  |  |  |  |  |  |  |  |
| Lambda cyhalothrin | Benin | *An. gambiae s.l* | 0.03g/m^2^ CS | 3 months | 20.7 | 25.8 | 72.1 | 8.9 | [30] |
|  |  | *An. gambiae* | 0.03g/m^2^ CS | 6 months | 50.0 | 8.8 | 8.8 | - | [71] |
|  | South Africa | *An. arabiensis* | 0.03g/m^2^ CS | 5 months | 56.3 | 39.0 | 48.0 | - | [88] **^** |
|  | Tanzania | *An. gambiae & An. funestus* | 0.03g/m^2^ CS | 7 months | 71.7 | 59.0 | - | - | [87] |
|  |  |  |  |  |  |  |  |  |  |
| Bendiocarb | Benin | *An. gambiae s.s* | 0.02g/ m^2^ | 2 Months | 20.8 | 87.5 | 92.9 | 10.0 | [53] |

**^¢^** This table includes a section of studies conducted in Africa, in areas where no resistance against DDT or pyrethroids had been reported. In studies where parameter values were not explicitly stated in the original publication, these values have been calculated from summary tables given in those original publications. ***Deterrence*** is calculated as the difference between number of mosquitoes entering treated huts and number entering control huts and is presented as a percentage of the number entering the control hut. ***Feeding inhibition*** is calculated as the percentage of all mosquitoes entering the treated huts that do not manage to feed and ***toxicity***, as the percentage of mosquitoes entering the treated hut that die. ***Excess exit*** is derived as the difference between percentage exit rates in sprayed and unsprayed huts, based on values presented in the original publications. The column for ***duration*** refers to the period after spraying, for which the data included in the analysis was collected.

**^** Studies by Service *et al* 1964 [52] and Sharp *et al* 1990 [51] were conducted in local houses fitted with exit traps, unlike in all the other studies where specially designed experimental huts were used.

.

***** Only mosquitoes collected from the floors are included in this row

**^+^** The formula used by Smith and Webley [86] , to calculate deterrence is slightly different from that used in the other publications. That is, instead of using parallel catches in control huts as the reference, deterrence is determined by comparing number of mosquitoes entering treated huts with an expected number (N), which is calculated as N= (C x E)/C_1_, where C is the number of mosquitoes entering control hut after spraying, E is the number entering treated hut after spraying and C_1_ is the number entering control hut prior to the spraying of any hut. Also the results presented here are averages for all the months during which the experiments were conducted and may not exactly match the summary values in the original publication. For example, it should be noted that the deterrency value stated in the original publication is 60-70% which excludes the first month of the study.

**^^**The study on the carbamate, Bendiocarb, was conducted in an area with high frequency of pyrethroid resistance, but with no resistance against the carbamates themselves [53], thus permitting its inclusion in this review, which otherwise considered only studies in areas where mosquitoes were susceptible to DDT and pyrethroids.

**^r^**All references are continuous with the list in the main article
