# Supplementary material for: Combining indoor residual spraying and insecticide-treated nets for malaria control in Africa: a review of possible outcomes and an outline of suggestions for the future
Source: Malar J. 2011 Jul 28;10:208. doi: 10.1186/1475-2875-10-208 (PMC3155911; doi:10.1186/1475-2875-10-208)
Supplement: Additional file 2 — Properties of insecticides commonly used in ordinary home-treated ITNs. A table showing properties of conventionally treated nets (ordinary home-treated ITNs) commonly used in Africa, on mosquitoes that enter or those that attempt to enter human huts. The effects are classified as deterrence, feeding inhibition, toxicity, and excess exit. The nets are grouped as per the active ingredients (insecticides) used to treat them. [file 1475-2875-10-208-S2.DOC]

**Table S2:** Properties of conventionally treated nets (ordinary home-treated ITNs) commonly used in Africa, on mosquitoes that enter or those that attempt to enter human huts. The effects are classified as deterrence, feeding inhibition, toxicity, and excess exit **^¢^**. The nets are grouped as per the active ingredients (insecticides) used to treat them.

| **Insecticide** | **Country** | **Major Vector** | **Washing** | **Dosage** | **Holes** | **Deterrence (%)** | **Feeding inhibition (%)** | **Toxicity (%)** | **Excess % exit** | **Reference^r^** |
| --- | --- | --- | --- | --- | --- | --- | --- | --- | --- | --- |
| Alpha cypermethrin | The Gambia | *An. gambiae s.l* | Unwashed | 100mg/m^2^ | Yes | 0 | 92.0 | 94.0 | - | [78] |
|  |  |  | Washed | 100mg/m^2^ | Yes | 0 | 91.0 | 74.0 | - |  |
|  | Tanzania | *An. arabiensis* | Unwashed | 25mg/m^2^ | Yes | 25.0 | 82.6 | 32.8 | 1.9 | [72] **^δ^** |
|  |  | *An. gambiae & An funestus* | Unwashed | 10mg/m^2^ | Yes | 45.8 | 81.5 | 59.5 | - | [96] |
|  |  |  | Washed | 10mg/m^2^ | Yes | 27.9 | 67.7 | 24.8 | - |  |
|  |  |  | Unwashed | 20mg/m^2^ | Yes | 21.2 | 68.5 | 63.4 | - |  |
|  |  |  | Washed | 20mg/m^2^ | Yes | 13.6 | 66.7 | 43.5 | - |  |
|  |  |  | Unwashed | 40mg/m^2^ | Yes | 11.4 | 79.1 | 50.1 | - |  |
|  |  |  | Washed | 40mg/m^2^ | Yes | 44.2 | 79.2 | 43.5 | - |  |
|  |  | *An. gambiae* | Unwashed | 20mg/m^2^ | Yes | 21.1 | 67.9 | 72.0 | 3.5 | [74] |
|  |  | *An funestus* | Unwashed | 20mg/m^2^ | Yes | 32.7 | 69.3 | 70.6 | 8.4 |  |
|  |  | *An. gambiae* | Washed | 20mg/m^2^ | Yes | 0 | 29.9 | 69.6 | 6.0 |  |
|  |  | *An funestus* | Washed | 20mg/m^2^ | Yes | 7.7 | 9.9 | 58.4 | 4.8 |  |
|  |  |  |  |  |  |  |  |  |  |  |
| Permethrin | Tanzania | *An. arabiensis* | Unwashed | 200mg/m^2^ | Yes | 33.7 | 72.0 | 49.8 | - | [75] ***^+^*** |
|  |  |  | Unwashed | 200mg/m^2^ | No | 20.6 | 61.0 | 41.9 | - |  |
|  |  |  | Unwashed | 80mg/m^2^ | No | 10.6 | 71.2 | - | 28.3 | [76] **^C^** |
|  |  | *An. arabiensis* | Unwashed | 25mg/m^2^ | Yes | 35.3 | 85.8 | 15.2 | 5.9 | [72] |
|  |  |  | Unwashed | 200mg/m^2^ | No | 57.1 | 75.0 | 89.0 | 27.0 | [76] |
|  |  |  | Unwashed | 1000mg/m^2^ | No | 66.6 | 63.0 | 70.0 | 56.0 |  |
|  |  | *An. gambiae & An funestus* | Unwashed | 200mg/m^2^ | Yes | 38.7 | 97.8 | 46.3 | - | [73] |
|  |  |  | Unwashed | 200mg/m^2^ | Yes | 20.5 | 82.2 | 29.8 | - |  |
|  | Kenya | *An. gambiae* | Unwashed | 500mg/m^2^ | No | 15.0 | 83.9 | - | 50.8 | [97] **^γ^** |
|  |  | *An. arabiensis* | Unwashed | 500mg/m^2^ | No | 0 | 66.7 | - | 13.9 |  |
|  |  | *An. funestus* | Unwashed | 500mg/m^2^ | No | 35.7 | 85.9 | - | 49.6 |  |
|  |  | *An. gambiae s.s* | Unwashed | 500mg/m^2^ | No | 94.6 | - | - | - | [98] **^γ^** |
|  |  | *An. funestus.* | Unwashed | 500mg/m^2^ | No | 96.7 | - | - | - |  |
|  | The Gambia | *An. gambiae s.l.* | Unwashed | 5mg/m^2^ | Yes | 33.0 | 96.3 | 74.0 | 2.0 | [77] **^λ^** |
|  |  | *An. gambiae s.l.* | Unwashed | 50mg/m^2^ | Yes | 45.1 | 98.2 | 75.0 | 4.0 |  |
|  |  | *An. gambiae s.l.* | Unwashed | 500mg/m^2^ | Yes | 69.9 | 98.7 | 79.0 | 10.0 |  |
|  |  |  |  |  |  |  |  |  |  |  |
| Lambda Cyhalothrin | The Gambia | *An. gambiae s.l.* | Unwashed | 25mg/m^2^ | Yes | 33.3 | 97.8 | 89.0 | 0 | [77] **^λ^** |
|  | Tanzania | *An. gambiae & An funestus* | Unwashed | 10mg/m^2^ | Yes | 33.6 | 63.3 | 71.4 | - | [96] |
|  |  |  | Washed | 10mg/m^2^ | Yes | 31.8 | 54.8 | 61.3 | - |  |
|  |  |  | Unwashed | 20mg/m^2^ | Yes | 32.6 | 63.3 | 74.8 | - |  |
|  |  |  | Washed | 20mg/m^2^ | Yes | 23.0 | 62.3 | 56.0 | - |  |
|  |  | *An. gambiae s.l.* | Unwashed | 18mg/m^2^ | Yes | 26.4 | 96.1 | 98.5 | 10.7 | [30] |
|  |  |  |  |  |  |  |  |  |  |  |
| Deltamethrin | The Gambia | *An. gambiae s.l* | Unwashed | 25mg/m^2^ | Yes | 11 | 93 | 88 | - | [78] |
|  |  |  | Washed | 25mg/m^2^ | Yes | - | 87 | 74 | - |  |
|  |  |  | Unwashed | 500mg/m^2^ | Yes | 60 | 98 | 72 | - |  |
|  |  |  | Washed | 500mg/m^2^ | Yes | - | 87 | 54 | - |  |
|  |  |  | Unwashed | 25mg/m^2^ | Yes | 22 | 98 | 86 | - |  |
|  |  |  | Washed | 25mg/m^2^ | Yes | 0 | 87 | 87 | - |  |
|  | Tanzania | *An. arabiensis* | Unwashed | 25mg/m^2^ | Yes | 30.7 | 81.4 | 33.0 | 2.5 | [72] |
|  |  | *An. gambiae* | Washed | 25mg/m^2^ | Yes | 22.5 | 89.0 | 69.0 | 6 | [69] |
|  |  |  | Unwashed | 25mg/m^2^ | No | 0 | 90.3 | 83.9 | - | [70] |
|  |  |  | Washed | 25mg/m^2^ | No | 0 | 91.2 | 70.2 | - |  |
|  |  | *An. gambiae & An funestus* | Washed | 25mg/m^2^ | No | 0 | 95.2 | 88.0 | - |  |

^¢^ This table includes a section of studies conducted in Africa, in areas where no resistance against DDT or pyrethroids had been reported. In studies where parameter values were not explicitly stated in the original publication, these values have been calculated from summary tables given in those original publications. ***Deterrence*** is calculated as the difference between number of mosquitoes entering treated huts and number entering control huts and is presented as a percentage of the number entering the control hut. ***Feeding inhibition*** is calculated as the percentage of all mosquitoes entering the treated huts that do not manage to feed. For purposes of uniformity, this formula was also applied to recalculate feeding inhibition for those studies where the authors had originally corrected the percentage feeding rates in treatment huts on the basis of feeding rates in control huts e.g. in Tungu *et al*., 2010 [69]. ***Toxicity*** on the other hand has been calculated as the percentage of mosquitoes entering the treated hut that die and ***excess exit*** is derived as the difference between percentage exit rates in sprayed and unsprayed huts, based on values presented in the original publications.

**^δ^** In the study by Mosha *et al* 2008 [72], the percentage mortality observed among mosquitoes collected in control huts was greater than 20%, therefore the toxicity values represented here are statistically corrected percentages.

*^+^* In studies by Lines *et al* 1985 and Lines *et al* 1987, the vector species are reported as *An. gambiae s.l.* though the original publications also had statements indicating that these mosquito populations were almost entirely *An. arabiensis* [75, 76].

**^C^** Results represented in this raw from the study by Lines *et al* [76] were obtained from tests of nets made of cotton rather than polyester as used in the rest of the studies

**^λ^** Deterrency and feeding rates in the Lindsay *et al.,* 1991 paper were recalculated, by subjecting the log numbers presented in the original publication to a microsoft excel function (z = IMEXP) that returns the actual number of mosquitoes (z) as an exponential of complex numbers originally in x + y_i_ or x + y_j_ format.

**^γ^** In the studies by Mathenge *et al.,* 2001[97] and Bogh *et al*., 1998[98], the data used was based on pyrethrum spray catches done inside local huts and also from catches of exiting mosquitoes trapped using Colombian curtains [57] installed around village huts that were allocated (or not allocated) nets.

**^r^**All references are continuous with the list in the main article
