# Supplementary material for: Combining indoor residual spraying and insecticide-treated nets for malaria control in Africa: a review of possible outcomes and an outline of suggestions for the future
Source: Malar J. 2011 Jul 28;10:208. doi: 10.1186/1475-2875-10-208 (PMC3155911; doi:10.1186/1475-2875-10-208)
Supplement: Additional file 3 — Properties of different long lasting insecticidal nets (LLINs) commonly used in Africa. A table showing properties of different long lasting insecticidal nets (LLINs) commonly used in Africa, on mosquitoes that enter or those that attempt to enter human occupied huts. The effects are classified as deterrence, feeding inhibition, toxicity, and excess exit. [file 1475-2875-10-208-S3.DOC]

**Table S3:** Properties of different long lasting insecticidal nets (LLINs) commonly used in Africa, on mosquitoes that enter or those that attempt to enter human occupied huts. The effects are classified as deterrence, feeding inhibition, toxicity, and excess exit **^¢^**.

| ***Type*** *^¥^* | ***Insecticide*** | ***Country*** | ***Major Vector*** | ***Washing*** | ***Holes*** | **Deterrence (%)** | **Feeding inhibition (%)** | **Toxicity (%)** | **Excess % exit** | **Reference^r^** |
| --- | --- | --- | --- | --- | --- | --- | --- | --- | --- | --- |
| PermaNet 2.0™ | Deltamethrin | Tanzania | *An. gambiae* | Unwashed | Yes | 20.6 | 90.0 | 95.0 | 0 | [69] |
|  |  |  |  | Washed | Yes | 18.9 | 91.0 | 85.0 | 2 |  |
|  |  |  | *An. gambiae* | Unwashed | No | 0 | 93.0 | 97.7 | - | [70] |
|  |  |  |  | Washed | No | 0 | 96.4 | 96.6 | - |  |
|  |  |  | *An. gambiae & An funestus* | Unwashed | No | 0 | 93.4 | 85.5 | - | [70] |
|  |  |  |  | Washed | No | 0 | 98.2 | 93.0 | - |  |
|  |  |  |  |  |  |  |  |  |  |  |
| PermaNet 3.0™ | Deltamethrin | Tanzania | *An. gambiae* | Unwashed | Yes | 41.2 | 97.0 | 95.0 | 0 | [69] |
|  |  |  |  | Washed | Yes | 22.8 | 90.0 | 94.0 | 0 |  |
|  |  |  |  |  |  |  |  |  |  |  |
| Interceptor™ | Alpha cypermethrin | Benin | *An. gambiae s.l* | Unwashed | No | 22.5 | 90.0 | 95.0 | 22.5 | [71] **^ß^** |
|  |  |  |  | Washed | No | 22.5 | 90.0 | 95.0 | 22.5 |  |
|  |  | Tanzania | *An. gambiae* | Unwashed | No | 0 | 88.0 | 93.0 | 15.0 |  |
|  |  |  |  | Washed | No | 0 | 82.0 | 73.0 | 15.0 |  |
|  |  |  | *An. funestus* | Unwashed | No | 0 | - | 76.0 | - |  |
|  |  |  |  | Washed | No | 0 | 86.0 | 60.0 | - |  |
|  |  |  | *An. gambiae s.l.* | Unwashed | No | - | 93.0 | 88.0 | - |  |
|  |  |  |  | Washed | No | - | 79.0 | 84.0 | - |  |
|  |  |  | *An. funestus* | Unwashed | No | - | 67.0 | - | - |  |
|  |  |  |  | Washed | No | - | 61.0 | 96.0 | - |  |
|  |  |  |  |  |  |  |  |  |  |  |
| Olyset™ | Permethrin | Tanzania | *An. arabiensis* | Unwashed | Yes | 0 | 96.3 | 11.8 | 25.6 | [72] **^δ^** |
|  |  |  | *An. gambiae & An funestus* | Unwashed | No | 5.4 | 87.2 | 56.0 | - | [73] |
|  |  |  |  | Unwashed | No | 0 | 90.3 | 55.0 | - |  |
|  |  |  |  | Washed | No | 0 | 97.2 | 70.0 | - |  |
|  |  |  |  | Unwashed | No | 0 | 80.4 | 49.0 | - |  |
|  |  |  | *An. gambiae* | Unwashed | Yes | 0 | 40.9 | 62.7 | 7.2 | [74] |
|  |  |  | *An funestus* | Unwashed | Yes | 28.9 | 49.9 | 73.9 | 1.4 |  |
|  |  |  | *An. gambiae & An funestus* | Washed | No | 0 | 81.1 | 57.5 | - | [73] **^µ^** |
|  |  |  | *An. gambiae* | Washed | Yes | 0 | 0 | 40.0 | 5.9 | [74] **^µ^** |
|  |  |  | *An funestus* | Washed | Yes | 30.8 | 0 | 58.9 | 4.2 |  |

^¢^ This table includes a section of studies conducted in Africa, in areas where no resistance against DDT or pyrethroids had been reported. In studies where parameter values were not explicitly stated in the original publication, these values have been calculated from summary tables given in those original publications. ***Deterrence*** is calculated as the difference between number of mosquitoes entering treated huts and number entering control huts and is presented as a percentage of the number entering the control hut. ***Feeding inhibition*** is calculated as the percentage of all mosquitoes entering the treated huts that do not manage to feed. For purposes of uniformity, this formula was also applied to recalculate feeding inhibition for those studies where the authors had originally corrected the percentage feeding rates in treatment huts on the basis of feeding rates in control huts e.g. in Tungu *et al*., 2010 [69]. ***Toxicity*** on the other hand has been calculated as the percentage of mosquitoes entering the treated hut that die and ***excess exit*** is derived as the difference between percentage exit rates in sprayed and unsprayed huts, based on values presented in the original publications.

*^¥^* **PermaNet 2.0™** is a 00% polyester LLIN coated with 55-62mg of synthetic deltamethrin per square metre. **PermaNet 3.0™** on the other hand is a mosaic-style LLIN specifically designed for the control of insecticide resistant mosquito populations. Its side panels, which unlike PermaNet 2.0™ have strengthened borders, are made of deltamethrin-coated-polyester (with approximately 118 mg/m^2^ of deltamethrin), while the top panel is made of monofilament polyethylene fabric into which a higher dose of deltamethrin (approx. 180 mg/m^2^) and approximately 1100mg/m^2^ of a synergist, piperonyl butoxide (PBO) are incorporated. This synergist inhibits mixed function oxidases, which are known to be associated with pyrethroid resistance. PermaNet 3.0™ is also manufactured by Vestergaard Frandsen, Denmark. **Interceptor™** is a long lasting insecticidal net made of polyester coated with alpha cypermethrin (200mg/m^2^). It is manufactured by BASF, Germany. Finally, **Olyset™** is made of a polyethylene netting (150 deniers), that is impregnated during manufacture with synthetic permethrin at a concentration of 2% (equivalent to 1000mg of active ingredient per square metre). It is manufactured by A to Z company, Tanzania.

**^ß^** The results for Interceptor™ nets evaluation in Benin are reported in the WHO report in very general terms as follows: high mortality (above 95%), high blood feeding inhibition (above 90%), 15-30% deterrence and 10-35% increase in exophilly [71]. Values reported in this table are therefore estimated as minimum mortality (95%) minimum feeding inhibition (90%), mean deterrence (22.5%) and mean excess exit (22.5%).

**^δ^** In the study by Mosha *et al* 2008 [72], the percentage mortality observed among mosquitoes collected in control huts was greater than 20%, therefore the toxicity values represented here are statistically corrected percentages.

**^µ^** The data represented in these specific rows were collected from studies where the Olyset™ nets tested had already been in use for 4 years [73] or 7 years [74].

**^r^**All references are continuous with the list in the main article
